# Characteristics of Optic Neuritis in South Korean Children and Adolescents: A Retrospective Multicenter Study

**DOI:** 10.1155/2022/4281772

**Published:** 2022-09-07

**Authors:** Kyung-Ah Park, Hee Kyung Yang, Jinu Han, Seong-Joon Kim, Sung Eun Park, Haeng-Jin Lee, Sueng-Han Han, Sei Yeul Oh, Jeong-Min Hwang

**Affiliations:** ^1^Department of Ophthalmology, Samsung Medical Center, Sungkyunkwan University School of Medicine, Seoul, Republic of Korea; ^2^Department of Ophthalmology, Seoul National University College of Medicine, Seoul National University Bundang Hospital, Seongnam, Republic of Korea; ^3^Institute of Vision Research, Gangnam Severance Hospital, Department of Ophthalmology, Yonsei University College of Medicine, Seoul, Republic of Korea; ^4^Department of Ophthalmology, Seoul National University College of Medicine, Seoul National University Hospital, Seoul, Republic of Korea; ^5^Institute of Vision Research, Severance Hospital, Department of Ophthalmology, Yonsei University College of Medicine, Seoul, Republic of Korea; ^6^Department of Ophthalmology, Jeonbuk National University College of Medicine, Jeonju, Republic of Korea

## Abstract

**Purpose:**

To analyze the clinical characteristics and prognosis of optic neuritis (ON) in pediatric patients aged <19 years in South Korea.

**Methods:**

This multicenter retrospective cohort study included 127 pediatric patients (median age: 10.3 (IQR: 7.3–14.2) years; female, 62.2%) who experienced ON for the first time between January 2004 and January 2018, with data obtained from five tertiary university-based hospitals in Korea. When ON was bilateral, the worse eye was selected for analysis. The baseline clinical characteristics and prognoses of patients, as well as the associations between these parameters, were analyzed.

**Results:**

The baseline clinical characteristics of the patients were as follows: best-corrected visual acuity (BCVA) < 20/200, 65.9%; pain on eye movement, 47.2%; optic disc swelling, 66.9%; and bilateral involvement, 41.7%. Among 101 patients who were followed up for ≥6 months, 48 (47.5%), 12 (11.9%), 19 (18.8%), 13 (12.9%), and 9 (8.9%) had been diagnosed with isolated ON, recurrent ON, multiple sclerosis (MS), neuromyelitis optica spectrum disorder (NMOSD), and acute disseminated encephalomyelitis (ADEM)-related ON, respectively. At the latest visit, 81.9% and 71.1% had achieved BCVA of ≥20/40 and ≥ 20/25, respectively. Only disc swelling at presentation was associated with poor baseline BCVA (coefficient: 0.31, *P*=0.004) and greater improvement in BCVA (coefficient: 0.49, *P* = 0.001*P*=0.001); there were no significant associations between the baseline factors and final BCVA.

**Conclusions:**

This study demonstrated pediatric ON-related clinical characteristics and visual outcomes in South Korea. Within this cohort, in about 40.6% of patients, ON was associated with other demyelinating diseases, namely, MS, NMOSD, and ADEM.

## 1. Introduction

Optic nerve inflammation has various causes, including infectious, granulomatous, paraneoplastic, or inflammatory demyelinating etiologies. Early determination of the cause of optic neuritis (ON) is an important aspect of providing appropriate treatment [1]. Demyelinating ON most commonly affects young adults. However, ON is less prevalent among children than it is among adults [2]. According to a population-based study, the annual incidence of ON among pediatric patients in South Korea is 1.04 per 100,000 people [3], which is less than that among adults (2.21–3.29 per 100,000) [4, 5]. This is also slightly lower than the incidence of pediatric ON in Taiwan (1.79–2.46 per 100,000) [6] and higher than that in the US [7]. In terms of clinical manifestation, ON in children is reported to show more distinct features than in adults [8–24]. Children are likely to have a preceding viral illness, painless bilateral optic nerve swelling, and severe vision loss at presentation [8–24]. However, most available information about pediatric ON is based on retrospective studies with relatively small samples (up to 102) [8, 9, 15, 23, 25–27]. The Optic Neuritis Treatment Trial was performed on adults aged 18–46 years old, and because 85% of them were Caucasian [28], its findings cannot be generalized to Asian pediatric patients [28]. It has also been reported that the clinical features of patients with ON differ among different ethnicities in both adults [29] and children [30]. A multicenter prospective study on pediatric ON was conducted in the US, but only 9% of the included children were of Asian ethnicity [27]. In addition, large-scale studies on pediatric ON are rare in Asia [8, 9, 25–27]. To address these gaps in the literature, we conducted a multicenter study to investigate the clinical characteristics and prognoses of pediatric patients with ON in South Korea. We also analyzed prognostic factors associated with the visual outcomes of ON.

## 2. Methods

This multicenter retrospective case series included pediatric patients with ON from five tertiary university-based hospitals in Seoul and the Gyeongi province: Bundang Seoul National University Hospital, *n* = 38; Gangnam Severance Hospital, *n* = 2; Samsung Medical Center, *n* = 28; Seoul National University Hospital, *n* = 36; and Shinchon Severance Hospital, *n* = 23. All patients were aged <19 years at the time of the first presentation of ON between January 2004 and January 2018. Approval was obtained from the appropriate institutional review boards at individual centers.

The main inclusion criteria were as follows: (1) Diagnosis of ON in at least one eye based on clinical symptoms such as visual loss with or without pain on eye movement, (2) presence of relative afferent pupillary defect (RAPD) in the case of unilateral involvement, (3) presence of at least one of the following features in the affected eye: BCVA deficit that is at least two lines below the age-based norms; color vision reduction; visual field defect; or optic disc swelling. The exclusion criteria were as follows: evidence of metabolic, toxic, inherited, mitochondrial, vascular, infectious, or compressive etiology affecting the optic nerve; intracranial hypertension; clinical evidence indicating mitochondrial disorders; or previously diagnosed amblyopia [31]. Patients were excluded if they had pre-existing ocular abnormalities or previous episodes of ON.

Data on demographic characteristics such as sex, age at onset, history of vaccination, the occurrence of infection within one month before the onset of ON, BCVA at presentation, time to first recurrence from the onset of ON, bilateral optic nerve involvement, the number of recurrences, the presence or absence of RAPD, pain on eye movement, optic disc swelling at presentation, white matter lesions (WMLs) on magnetic resonance (MR) images, and enhancement of the optic nerve on MR images were obtained. Serologic findings, including those of blood and cerebrospinal fluid (CSF) analyses, erythrocyte sedimentation rate, C-reactive protein, aquaporin-4 (AQP4) antibody, myelin oligodendrocyte glycoprotein (MOG) antibody, and anti-nuclear antibody, were also recorded. Serum levels of AQP4 and MOG antibodies were tested using a live cell-based assay [32, 33]. When ON was bilateral, the worse eye was selected for the analysis.

The final diagnoses were confirmed based on clinical and radiological information related to one of the following diagnostic categories: (1) isolated ON, (2) recurrent ON, and (3) multiple sclerosis (MS), in accordance with the 2013 International Pediatric Multiple Sclerosis Study Group consensus criteria [34], (4) neuromyelitis optica (NMO) spectrum disorder (NMOSD), in accordance with the 2015 International Panel for NMO diagnosis criteria, and (5) acute disseminated encephalomyelitis (ADEM) and ADEM-ON, in accordance with the 2013 International Pediatric Multiple Sclerosis Study Group consensus criteria [34].

Regression analyses were used to determine whether there were any associations between baseline characteristics and visual outcomes. We considered the following baseline factors: age at presentation, sex, disc swelling, final diagnosis (neurological associated, i.e., ADEM, NMO, or MS vs. isolated or recurrent ON), bilaterality, and the presence of WMLs on MR images. The visual outcomes were categorized as follows: good, the latest BCVA ≥20/40 with no visual field defect; fair, 20/200≤ BCVA <20/40 or ≥20/40 with permanent visual field defect; and poor, BCVA <20/200 [11, 35, 36]. Statistical analyses were conducted using Stata v.16.1 (StataCorp LLC, College Station, Texas, USA). Continuous variables were analyzed using the Kruskal-Wallis test. Categorical data were compared using the chi-square or Fisher's exact test. *P* values of <0.05 were considered to be statistically significant.

## 3. Results

### 3.1. Baseline Characteristics

**Figure 1:**
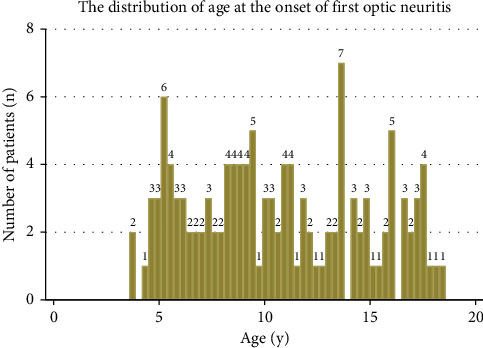
Age distribution of pediatric patients with optic neuritis at presentation.

In total, 127 children (62.2% female) who experienced the first occurrence of ON were included. The median age at presentation was 10.3 years (interquartile range [IQR], 7.3–14.2 years, Figure 1). The demographic and clinical features are listed in Table 1. The mean baseline BCVA was 1.26 logMAR (IQR, 0.82–1.7, range 0–1.7). Eighty-three patients (65.9%) presented with a BCVA <20/200 at the initial visit. Eye pain on movement was noted in 51 of the 108 (47.2%) patients, and disc swelling was noted in 83 of the 124 (66.9%) patients. Four patients had previous ADEM episodes, and the diagnosis of MS was made in one patient before the first episode of ON. Thirty-nine (36.1%) of the 109 children had a previous febrile illness within 1 month of the occurrence of ON. Seven (6.9%) of the 101 children had a vaccination history within 1 month of ON occurrence, and none of these seven patients were diagnosed with ADEM-ON. Because testing for AQP4 and MOG antibodies was not available during early study periods, the former and latter were tested in 58 (45.7%) and 9 (7.1%) patients, respectively. Among these patients, seven patients (12.1%) and one patient (who was included in the isolated ON group in this study) (11.1%) tested positive for AQP4 and MOG, respectively. The anti-nuclear antibody was tested in 90 patients, 21 (23.3%) of whom tested positive. The CSF analyses were available for 69 children, and intrathecal oligoclonal bands (OCBs) and pleocytosis were respectively noted in three (4.4%) and six (8.7%) children. All three patients who tested positive for intrathecal OCBs were subsequently diagnosed with MS. Twenty-nine patients (22.1%) experienced recurrence of ON during the follow-up period. The mean duration from the onset of ON to the first recurrence was 24.7 ± 39.2 months (range, 1–149 months).

Brain MR images of 126 patients (99.2%) were available at the initial visit. Optic nerve enhancement was detected in 102 patients (81.0%). The WMLs on MR images were noted in 41 (32.5%) patients at the time of presentation. The McDonald 2010 criteria and consensus definitions for pediatric MS, for dissemination in space and time, were met in 19 patients with subsequent follow-up MR images [37, 38]. The first treatment of ON was performed with 30 mg/kg or 1 g/day intravenous methylprednisolone for 3 days (*n* = 104, 81.9%) or 4–5 days (*n* = 16, 12.6%); seven patients (5.5%) received no treatment. Among the patients who received intravenous steroid treatment, 66 children (55%) received a more extended slow tapering of oral steroids beyond 2 weeks.

The automated visual field test was available to 111 patients at the time of initial visit. The most frequent pattern of visual field defect was central scotoma (*n* = 41, 36.9%), followed in descending order by generalized depression (*n* = 29, 26.1%), generalized constriction (*n* = 20, 18.0%), cecocentral scotoma (*n* = 11, 9.9%), and altitudinal defect (*n* = 10, 9.0%).

### 3.2. Disease Groups

The median duration of follow-up was 24 months (IQR, 6–51). A total of 111, 101, and 84 patients were followed up for ≥3 months, ≥6 months, and ≥1 year, respectively. Among the 101 patients that were followed up for ≥6 months, the following diagnoses were noted: isolated ON (including one patient who tested positive for the MOG antibody and did not have any other abnormal neurologic finding except for ON), 48 (47.5%); recurrent ON, 12 (11.9%); MS, 19 (18.8%); NMOSD, 13 (12.9%); and ADEM-ON, 9 (8.9%). The proportion of female patients (83.3%) was highest in the NMOSD group, and the isolated ON group had an almost equal sex ratio (Table 2). The number of recurrences and durations of follow-ups were significantly different among these groups (*P* < 0.001 and 0.003, respectively).

### 3.3. Outcomes of Visual Acuity

BCVA data were available for 100 of the 101 patients who were followed up for ≥6 months. Among these, 76 patients (76.0%), 13 patients (13%), and 11 patients (11%) achieved good, fair, and poor visual outcomes, respectively, at the latest follow-up.

**Figure 2:**
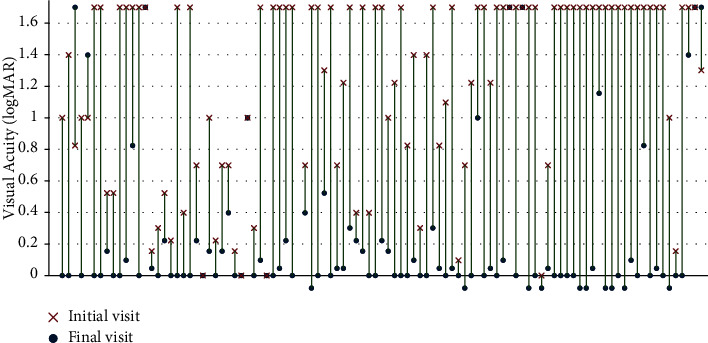
Paired dot plot showing initial and final best-corrected visual acuity (BCVA) in 100 patients with pediatric optic neuritis who were followed up for ≥6 months. BCVA presented as logMAR. BCVA improved from baseline to final visit by a mean value of 1.02 ± 0.70 logMAR (IQR, 0.30–1.7). BCVA of 20/800 or less was presented as 1.7 logMAR.

The mean baseline BCVA was 1.26 logMAR, which improved by a mean of 1.02 ± 0.70 logMAR (IQR, 0.30–1.7) to 0.22 ± 0.47 logMAR at the final visit (Figure 2). The mean final logMAR BCVAs in each group were as follows: isolated ON group, 0.16 ± 0.42; recurrent ON group, 0.34 ± 0.60; MS group, 0.12 ± 0.39; NMOSD group, 0.44 ± 0.62; and ADEM-ON group, 0.20 ± 0.48. The mean final logMAR BCVA was the worst in the NMOSD group, but there were no significant differences between the groups (*P*=0.250). The visual outcomes as categorical variables (good, fair, and poor) were not significantly different among the groups either (Table 2, *P*=0.720).

We analyzed the factors associated with a poor BCVA at presentation and improvement in BCVA. Only disc swelling at presentation was found to be associated with a poor BCVA at presentation (coefficient: 0.31, *P*=0.004) and an improvement in BCVA (coefficient: 0.49, *P*=0.001). Other factors such as age at presentation, sex, bilaterality, associated neurologic autoimmune diagnosis, and WMLs on MR images were not significantly associated with either of these two parameters. The data did not suggest a significant association between baseline characteristics and final BCVA.

## 4. Discussion

The current study, which included 127 children with ON from five tertiary centers who were treated over a 14-year period, is one of the largest multicenter studies conducted to date [8, 9, 15, 23, 25, 26]. The main findings of our study are as follows: (1) pain on eye movement was present in less than half of the pediatric patients (47.2%); (2) optic disc swelling was found in nearly two-thirds of pediatric patients (66.9%); (3) bilateral involvement was common (41.7%); (4) in a high percentage of pediatric patients (40.6%), ON was associated with other demyelinating diseases such as MS, NMOSD, and ADEM; (5) disc swelling was associated with both poor BCVA at presentation and greater improvement in BCVA; (6) there were no significant associations between baseline factors and final visual outcomes; and (7) NMOSD-related ON showed the highest rate of recurrence.

In our study, the overall clinical features of pediatric ON were similar to those reported in previous studies with Western and Asian children (Table 3) [8–24]. The median age at presentation in this study was 10.3 years, which is comparable to that in previous studies [8–24]. Moreover, the proportion of female patients (62.2%) along with the rates of optic disc swelling (66.9%) and bilateral involvement (41.7%) were higher, and the rate of pain on eye movement (47.2%) was relatively lower in our present study on pediatric patients than in previous studies on the adult population, and this finding is consistent with those of previous studies [8–24].

Optic nerve enhancement on MR images was detected in 81.0% of our patients. This feature is helpful for the clinical diagnosis of ON and is reported in up to 75–95% of adult patients with ON in Western countries [39–42]. This characteristic has been detected less frequently in Asian adults (30–74%) [43–46]. In a previous study on French children with ON, optic nerve enhancement was found in 29% (28 of 95) of the patients, which is much lower than that observed in our study [24]. These differences could be partly due to differences in the characteristics of the study subjects or the MR imaging protocols that were used in the study, or poor MR image quality for some young children. Moreover, WMLs on brain MR images were noted in 32.5% of the patients at presentation in our cohort, which was lower than the corresponding value of 45–52% reported in previous studies [24, 27]. In this regard, WMLs are closely associated with MS; the differences in the proportions of WMLs between studies may be related to the different proportions of patients with MS between the study cohorts.

Our study found that 18.8% and 12.9% of the patients who were followed up for ≥6 months had been diagnosed with MS and NMOSD, respectively. Although the rate of conversion to MS in our cohort (18.8%) was somewhat higher than that in previous Asian studies (4–10%) [8, 9, 15], it is still lower than that in prior studies conducted in Western countries (22–36%) [12, 22, 24]. The differences in the conversion rates to MS in adult patients with ON between Asian countries and Western countries have been well documented [47–49]. Similar differences may also be present in the pediatric population. The proportion of patients with NMOSD in this study was 12.9%, which was much higher than the corresponding value of 5–7% reported in Western counties [24, 27]. This could be related to the predisposition of non-Caucasians—especially Asians—to NMOSD [50–54]. Patients with NMOSD showed a higher rate of recurrence compared to patients with other etiologies.

We investigated factors associated with visual outcomes in pediatric patients with ON. The only factor associated with an improvement of BCVA and poor BCVA at presentation in this study was the presence of disc swelling at presentation. However, no other factors were significantly associated with final visual outcomes. Wan et al. previously analyzed the visual outcomes and associated factors in a relatively large cohort of 36 patients with ON with at least 1 year of follow-up [11]. They found that none of the clinical characteristics, such as sex, baseline vision, laterality, treatment, or underlying diagnosis, predicted poor visual outcomes, which is consistent with the findings of our study [11]. A recent prospective study on pediatric ON demonstrated that baseline BCVA was worse in younger patients with associated neurologic autoimmune diseases, those with WMLs on MR images, and those of non-White race and non-Hispanic ethnicity [27]. They also reported that there were no baseline factors associated with BCVA improvement. Their study did not analyze disc swelling. Based on our findings, we presume that pediatric patients with ON showing optic disc swelling, despite a relatively poor visual acuity at presentation, have a good prognosis that is comparable to that of patients without optic disc swelling.

This study has several limitations, including the retrospective design, variability of follow-up periods between patients, and unavailability of AQP4 and MOG antibody testing results in early study periods. Despite these limitations, our study provides important information on the baseline characteristics and visual outcomes of a relatively large number of Korean pediatric patients with ON. Although two-thirds of the patients experienced severe visual loss (<20/200) at presentation, 76% experienced good visual recovery at the final visit. Moreover, ON was associated with other neurological diseases (i.e., MS, NMOSD, and ADEM) in 40.6% of the pediatric patients.

In conclusion, our study is one of the largest multicenter studies summarizing the clinical characteristics and visual prognosis of pediatric ON. The baseline characteristics were similar to those reported in other countries. A relatively high proportion of pediatric ON showed optic disc swelling (67%), bilateral involvement (42%), and associations with other central nervous system demyelinating diseases (40%). NMOSD was found more frequently (12.9%) than it has been found in Western countries, showing the highest rate of recurrence among other etiologies. A more comprehensive future study investigating the seropositivity of MOG antibody testing in pediatric ON should extend our understanding of the clinical aspects of the disease in Koreans.

## Figures and Tables

**Table 1:** Clinical demographics of patients with pediatric onset optic neuritis.

	Numbers (total)	%, or SD (IQR)
Number of patients	127	100%
Sex (female)	79	62.2%

Age at onset (years)		
3–6	29	22.8%
7–9	32	25.2%
10–12	23	18.1%
≥13	43	33.9%
Mean (median)	10.7 (10.3)	4.1 (7.3–14.2)

Bilaterality	53 (127)	41.7%
Pain with eye movement	51 (108)	47.2%
Disc swelling at presentation	83 (124)	66.9%
Accompanying headache	37 (127)	29.1%
White matter lesions on MRI	41 (126)	32.5%
Preceding febrile illness within 1 month	39 (108)	36.1%
Vaccination history within 1 month	7 (101)	6.9%

Visual acuity at presentation	Total = 126	
≥20/20	4	3.2%
<20/20-≥ 20/40	11	8.7%
<20/40-≥ 20/200	28	22.2%
<20/200-> counting fingers	26	20.6%

Counting finger-no light perception	57	45.2%

Final visual outcome†	Total = 100	
Good	76	76%
Fair	15	15%
Poor	9	9%

SD = standard deviation, IQR = interquartile range. †Visual outcome was categorized as the latest BCVA ≥20/40 with no visual field defect (good), BCVA <20/40∼≥ 20/200 or ≥ 20/40 with permanent visual field defect (fair), and BCVA less than 20/200 (poor).

**Table 2:** Demographic, clinical, and serologic features of 101 children who were followed up for more than 6 months according to their demyelination syndrome diagnosis.

Variables	Isolated ON (*n* = 48)	Recurrent ON (*n* = 12)	MS (*n* = 19)	NMOSD (*n* = 13)	ADEM-ON (*n* = 9)	*P* value
Age, median (IQR), y	9.3 (6.7–13.3)	10.1 (6.0–13.9)	10.6 (7.8–14.1)	11.8 (9.5–13.8)	7.8 (5.4–11.5)	0.495
Female, *n* (%)	22 (45.8%)	8 (66.7%)	15 (79.0%)	10 (83.3%)	6 (66.7%)	0.247
Disc swelling at presentation	32/47 (68.1%)	7/12 (58.3%)	10/19 (52.6%)	6/12 (50%)	7/9 (77.8%)	0.381
Intrathecal OCBs	0/19 (0%)	0/5 (0%)	3/14 (17.7%)	0/12 (0%)	0/7 (0%)	0.121
Total no. of optic neuritis, median (IQR)	0 (0–0)	1 (1–1)	0 (0–1)	1 (0–1)	0 (0–0)	<0.001
Bilateral involvement	19 (39.6%)	6 (50.0%)	8 (42.1%)	7 (53.9%)	2 (22.2%)	0.788
MRI enhancement	37/47 (78.7%)	8/12 (66.7%)	16/19 (84.2%)	11/13 (84.6%)	8/9 (88.9%)	0.834

Serologic testing						
AQP4 antibody	0/15	0/7	0/11	6/12 (50%)	0/1	
MOG antibody	1/2	0/1	0/2	0/1	0/1 (0%)	
ANA antibody	8/31 (25.8%)	3/8 (37.5%)	1/14 (7.1%)	3/13 (23.1%)	1/7 (14.3%)	

Visual outcome (*n* = 100)†						0.720
Good	40 (83.3%)	8 (66.7%)	14 (73.7%)	7 (58.3%)	7 (77.8%)	
Fair	5 (10.4%)	2 (16.7%)	4 (21.0%)	3 (25.0%)	1 (11.1%)	
Poor	3 (6.3%)	2 (16.7%)	1 (5.3%)	2 (16.7%)	1 (11.1%)	

logMAR visual acuity at latest visit (*n* = 100)†‡	0.16 ± 0.42	0.34 ± 0.60	0.12 ± 0.39	0.44 ± 0.62	0.20 ± 0.48	0.250
Follow-up duration, median (IQR)	30 (15–42)	47 (24–73)	32 (18–78)	54 (21–68)	47 (23–61)	0.003

ADEM = acute disseminated encephalomyelitis; ANA = anti-nuclear antibody; AQP4 = aquaporin-4; IQR = interquartile range; OCB = oligoclonal bands; ON = optic neuritis; MOG = myelin oligodendrocyte glycoprotein; MS = multiple sclerosis; NMO = neuromyelitis optica; NMOSD = neuromyelitis optica spectrum disorder. The percentages and interquartile ranges (IQR) are presented in parentheses. Continuous variables were analyzed by the Kruskal-Wallis test. Chi-square test or Fisher's exact test was used for categorical variables. †Visual outcome was only analyzed among 100 patients who were followed up for more than 6 months and whose BCVA data were available. ‡Visual outcome was categorized as the latest BCVA ≥20/40 with no visual field defect (good), BCVA <20/40∼≥ 20/200 or ≥ 20/40 with permanent visual field defect (fair), and BCVA less than 20/200 (poor).

**Table 3:** Comparison of patient demographics and clinical characteristics of pediatric optic neuritis.

Variable	Pineles et al. (*n* = 44) USA	Wan et al. (*n* = 46) USA	Averseng-peaureaux et al. (*n* = 102) France	Ambika et al. (*n* = 78) India	Chen et al. (*n* = 59) China	Present study (*n* = 127) Korea
Age, years (range)	10.2 (3.9–13.5)	12.6 (3.9–18.ct8)	11†	11.8 (2–18)	12.3 (5–18)	10.7 (3.6–18.6)
Gender (female), %	41%	72%	66%	54%	69%	62%
Pain with eye movement	—	43%	49%	46%	83%	47%
Bilateral involvement	36%	41%	37%	50%	63%	42%
Optic disc swelling	75%	67%	52%	50%	64%	67%
Severe visual loss at presentation (<20/200)	52%	50%	48%	NA	45%	66%

Final visual outcome						
≥20/40	85%	90%	72%‡	71%	85%	76%
<20/40-≥ 20/200	11%	8%	NA	NA	4%	15%
<20/200	4%	3%	NA	NA	11%	9%

Final diagnosis						
Isolated ON	48%	48%	58%	89%	69%	48%
Recurrent ON	18% (MOGAD)	—	6%	—	10%	12%
MS	11%	39%	21%	6%	3%	19%
NMOSD	7%	7%	5%	—	15%	13%
ADEM-ON	16%	7%	—	5%	3%	8%

ADEM = acute disseminated encephalomyelitis; NMOSD = neuromyelitis optica spectrum disorder; MOGAD = myelin oligodendrocyte glycoprotein-associated disorder; MS = multiple sclerosis; ON = optic neuritis. †The age in this study was presented as median. ‡72% of the patients had complete visual recovery (complete visual recovery defined as the recovery of normal visual acuity (20/20), or prior visual acuity if it was abnormal before optic neuritis occurrence).

## Data Availability

The data that support the findings of this study are available from the corresponding author upon reasonable request.
